# The Soluble Guanylyl Cyclase Activator Bay 58-2667 Selectively Limits Cardiomyocyte Hypertrophy

**DOI:** 10.1371/journal.pone.0044481

**Published:** 2012-11-07

**Authors:** Jennifer C. Irvine, Virat Ganthavee, Jane E. Love, Amy E. Alexander, John D. Horowitz, Johannes-Peter Stasch, Barbara K. Kemp-Harper, Rebecca H. Ritchie

**Affiliations:** 1 Heart Failure Pharmacology, Baker IDI Heart & Diabetes Institute, Melbourne, Victoria, Australia; 2 Department of Pharmacology, Monash University, Clayton, Victoria, Australia; 3 Department of Medicine, Monash University, Clayton, Victoria, Australia; 4 Cardiology Unit, The Queen Elizabeth Hospital, Woodville South, South Australia, Australia; 5 Pharma Research Center, Bayer Schering Pharma AG, Wuppertal, Germany; Maastricht University, The Netherlands

## Abstract

**Background:**

Although evidence now suggests cGMP is a negative regulator of cardiac hypertrophy, the direct consequences of the soluble guanylyl cyclase (sGC) activator BAY 58-2667 on cardiac remodeling, independent of changes in hemodynamic load, has not been investigated. In the present study, we tested the hypothesis that the NO^•^-independent sGC activator BAY 58-2667 inhibits cardiomyocyte hypertrophy *in vitro*. Concomitant impact of BAY 58-2667 on cardiac fibroblast proliferation, and insights into potential mechanisms of action, were also sought. Results were compared to the sGC stimulator BAY 41-2272.

**Methods:**

Neonatal rat cardiomyocytes were incubated with endothelin-1 (ET_1_, 60nmol/L) in the presence and absence of BAY 41-2272 and BAY 58-2667 (0.01–0.3 µmol/L). Hypertrophic responses and its triggers, as well as cGMP signaling, were determined. The impact of both sGC ligands on basal and stimulated cardiac fibroblast proliferation *in vitro* was also determined.

**Results:**

We now demonstrate that BAY 58-2667 (0.01–0.3 µmol/L) elicited concentration-dependent antihypertrophic actions, inhibiting ET_1_-mediated increases in cardiomyocyte 2D area and *de novo* protein synthesis, as well as suppressing ET_1_-induced cardiomyocyte superoxide generation. This was accompanied by potent increases in cardiomyocyte cGMP accumulation and activity of its downstream signal, vasodilator-stimulated phosphoprotein (VASP), without elevating cardiomyocyte cAMP. In contrast, submicromolar concentrations of BAY 58-2667 had no effect on basal or stimulated cardiac fibroblast proliferation. Indeed, only at concentrations ≥10 µmol/L was inhibition of cardiac fibrosis seen *in vitro*. The effects of BAY 58-2667 in both cell types were mimicked by BAY 41-2272.

**Conclusions:**

Our results demonstrate that BAY 58-2667 elicits protective, cardiomyocyte-selective effects *in vitro*. These actions are associated with sGC activation and are evident in the absence of confounding hemodynamic factors, at low (submicromolar) concentrations. Thus this distinctive sGC ligand may potentially represent an alternative therapeutic approach for limiting myocardial hypertrophy.

## Introduction

Pathological cardiac hypertrophy is a common manifestation of many cardiovascular disorders including heart failure, hypertension, diabetic heart disease and remodeling following myocardial infarction, which develops initially as an adaptive growth response [1]. In due course however, prolonged hypertrophy progresses to a maladaptive state, with impairments in systolic and diastolic function, as well as coronary vasodilator reserve, leading to heart failure [2]. Current treatment options are largely based on inhibition of the renin-angiotensin system, yet these therapies tend to slow progression of the disease, rather than reversing it [3], and consequently the development of new therapeutic strategies is warranted.

**Figure 1:**
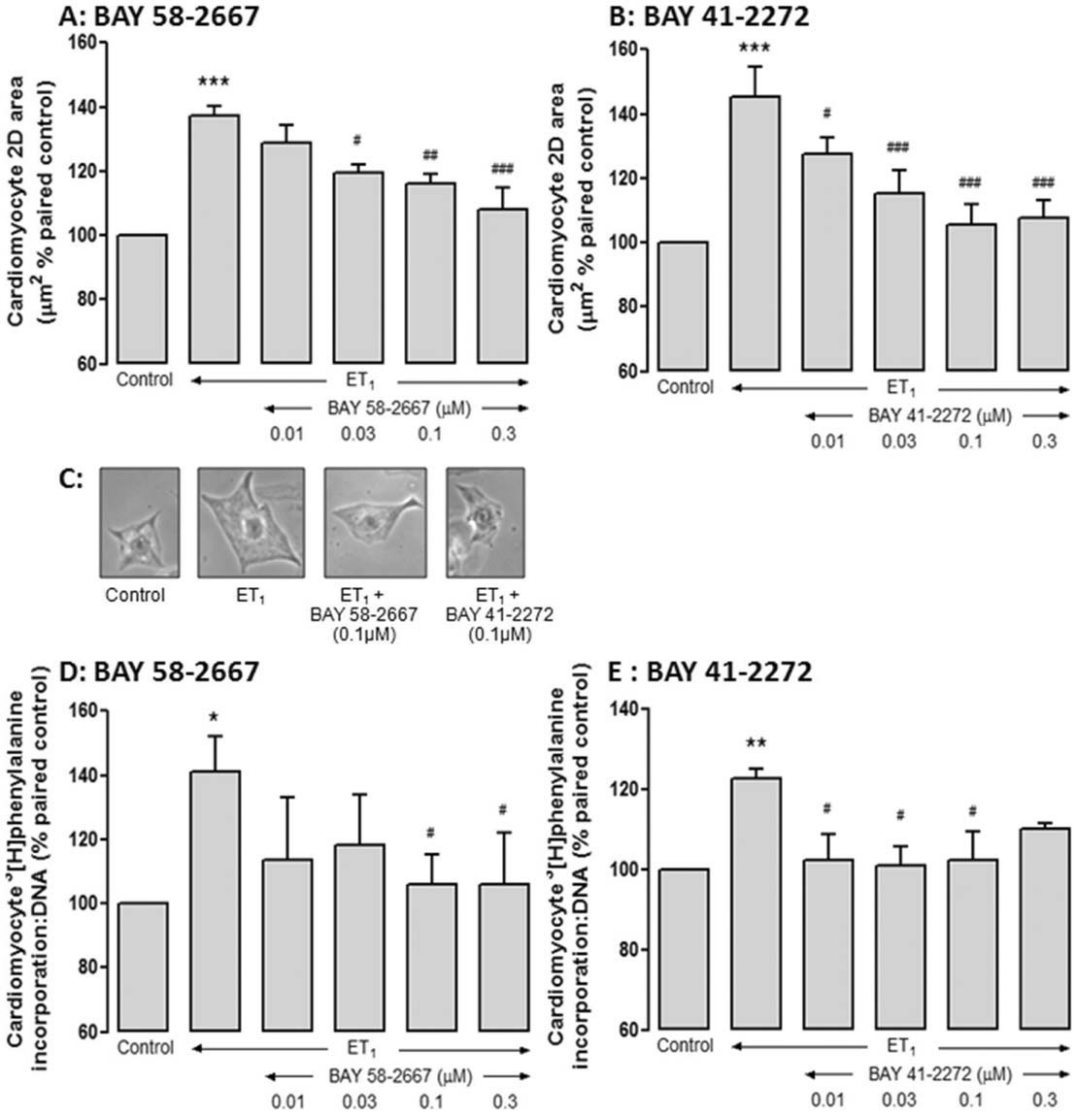
BAY 58-2667 elicits potent antihypertrophic actions. These actions are evident on **A** ET_1_ (60 nM)-induced cardiomyocyte size (0.01–0.3 µmol/L, n = 5 cardiomyocyte preparations); which was mimicked by **B** BAY 41-2272 (0.01–0.3 µmol/L, n = 4 cardiomyocyte preparations); **C** Representative results for cardiomyocyte size for each treatment studied (following 48 h of incubation), at 1034×1300 resolution and photographed at 10×magnification. **D** BAY 58-2667 similarly attenuated ET_1_ (60nmol/L)-induced cardiomyocyte *de novo* protein synthesis (BAY 58-2667, 0.01–0.3 µmol/L, n = 5 cardiomyocyte preparations); which was mimicked by **E** BAY 41-2272 (0.01–0.3 µmol/L, n = 4 cardiomyocyte preparations). *P<0.05, **P<0.01, ***P<0.001 vs control, ^#^P<0.05, ^##^P<0.01, ^###^P<0.001 vs ET_1_ alone (one-way repeated measures ANOVA with Bonferroni *post-hoc* analysis).

The ubiquitous second messenger cGMP has emerged as an exciting cardiovascular therapeutic target, attracting attention for the potential management of pulmonary hypertension, acute heart failure and cardiac necrosis and remodeling post myocardial infarction [4]–[6]. cGMP is formed from the catalytic conversion of GTP, by either the cytosolic sGC (the receptor for nitric oxide, NO^•^), or by the membrane-bound particulate GC isoform (pGC, activated by the natriuretic peptides ANP and BNP) [7]. Our work and others have indicated that cGMP is a powerful antihypertrophic mediator in the heart [8], [9]. These antihypertrophic actions of cGMP, evident across isolated cardiomyocytes to the intact heart, are mediated via cGMP-dependent protein kinase (cGK-I)-dependent signaling [10]–[14]; negative regulation of cardiomyocyte superoxide generation is a key mechanism of these actions [14], [15]. Chronic *in vivo* treatment with sGC ligands such as BAY 58-2667 (also known as cinaciguat) or BAY 41-2272 can limit hypertension-driven left ventricular hypertrophy in experimental models, at least using relatively high dosing strategies [16]–[20]. In each of these settings however, the antihypertrophic effect appeared secondary to the attenuation of hypertension via vasodilatation. The *direct* impact of selective sGC activation on cardiomyocyte hypertrophy however has not been investigated.

**Figure 2:**
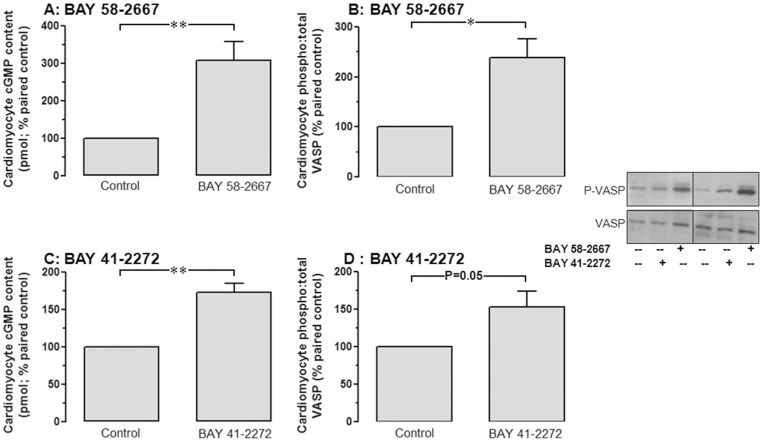
BAY 58-2667 activates cardiomyocyte cGMP/P-VASP signaling. BAY 58-2667 (0.1 µmol/L) increases cardiomyocyte **A** cGMP accumulation and **B** VASP phosphorylation. BAY 41-2272 (0.1 µmol/L) similarly increases cardiomyocyte **C** cGMP accumulation and **D** VASP phosphorylation. Representative Western blots are shown. *P<0.05, **P<0.01 vs control (Student's paired *t-*test).

Studies utilizing pharmacological and transgenic activation of cGK-I and cGMP-dependent phosphodiesterase inhibitors, as well as mouse models of BNP and pGC knockout (for review see [8], [21], [22]) suggest cGMP may also target the other contributor to cardiac remodeling, cardiac fibrosis. At least part of the antifibrotic actions of BNP in the heart may however be attributed to cGMP-independent actions via the NPR_C_ natriuretic peptide receptor [22]. Evidence specifically favoring cardiac antifibrotic actions with direct sGC ligands is however limited. Furthermore, this evidence fails to dissect out the direct actions of sGC at the cellular level, independent of confounding hemodynamic changes.

**Table 1:** The effect of BAY 58-2667 and BAY 41-2272 (both 0.1 µmol/L) on cardiomyocyte cAMP accumulation (expressed as percentage of paired controls, mean±SEM, both n = 6).

	Control	sGC ligand	DMSO vehicle	P
BAY 58-2667	100±0%	86±12%	91±6%	NS
BAY 41-2272	100±0%	78±9%	91±6%	NS (0.06)

In particular, the NO^•^-independent sGC activator BAY 58-2667 is thought to preferentially target Fe^3+^-oxidized or heme-free sGC, and may have an additive effect to NO^•^. This is in contrast to the NO^•^-independent sGC stimulator BAY 41-2272, that targets sGC in the Fe^2+^-heme-containing state and acts synergistically with NO^•^ [23]. BAY 58-2667 elicits potent vasodilator effects, unloading the heart. The extent to which this altered afterload mediates its associated anti-proliferative, anti-aggregatory and other effects has however not been previously investigated [16], [17], [24], [25]. The preference of BAY 58-2667 to target oxidized sGC potentially confers increased potency in the setting of disease [26]. The ability, however, of BAY 58-2667 to directly inhibit cardiac hypertrophy and/or fibrosis at the cellular level, independently of blood pressure lowering, has not yet been determined. Thus we tested the hypothesis that BAY 58-2667 elicits direct antihypertrophic effects in neonatal rat cardiomyocytes. Its efficacy on cardiac fibrosis *in vitro* was also determined. Our studies provide evidence that this sGC activator selectively and potently limit myocardial hypertrophy as a primary effect, independent of confounding hemodynamic changes.

**Figure 3:**
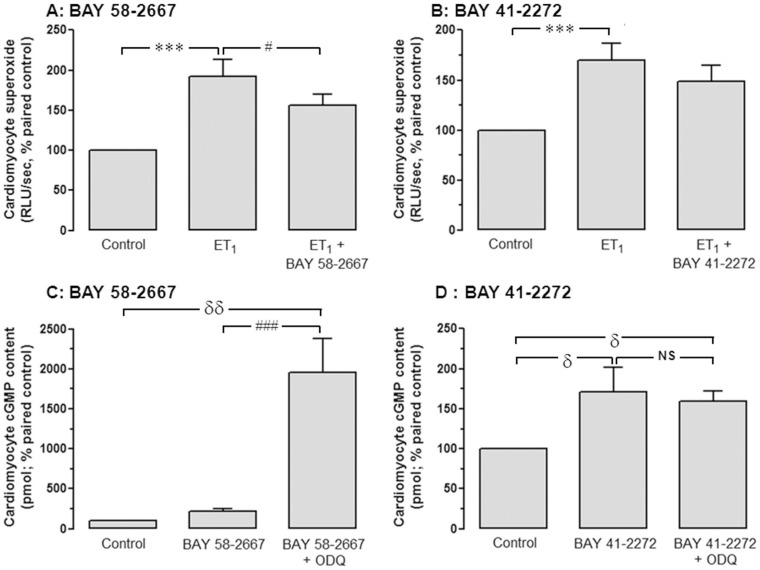
BAY 58-2667 suppresses cardiomyocyte ROS. The effects of **A** BAY 58-2667 and **B** BAY 41-2272 (both 0.1 µmol/L, n = 8 cardiomyocyte preparations) on cardiomyocyte superoxide generation, added 24 h prior to ET_1_ (60 nmol/L, determined on lucigenin-enhanced chemiluminescence). Values are expressed as percentage of paired control myocytes and given as mean±SEM, where n = number of myocyte preparations. The ability of sGC ligands to enhance cardiomyocyte cGMP accumulation in the presence of the sGC-oxidizing agent ODQ (10 µmol/L) is also shown, for **C** BAY 58-2667 and **D** BAY 41-2272 (both 0.1 µmol/L, n = 6 cardiomyocyte preparations). ***P<0.001, ^δδ^P<0.01, ^δ^P<0.05 vs control; ^###^P<0.001, ^#^P<0.05 vs BAY 58-2667 alone (one-way repeated measures ANOVA with Student-Newman-Keuls *post-hoc* analysis).

## Materials and Methods

This investigation conforms to both the *Guide for the Care and Use of Laboratory Animals* published by the US National Institutes of Health (NIH Publications No. 85–23, revised 1996) and the National Health and Medical Research Council of Australia guidelines, and was approved by the Animal Ethics Committee of the Alfred Medical Research and Education Precinct (AMREP; approval E/0698/2008/B). All materials were purchased from Sigma-Aldrich (St. Louis, USA) except where indicated, and were of analytic grade or higher.

**Figure 4:**
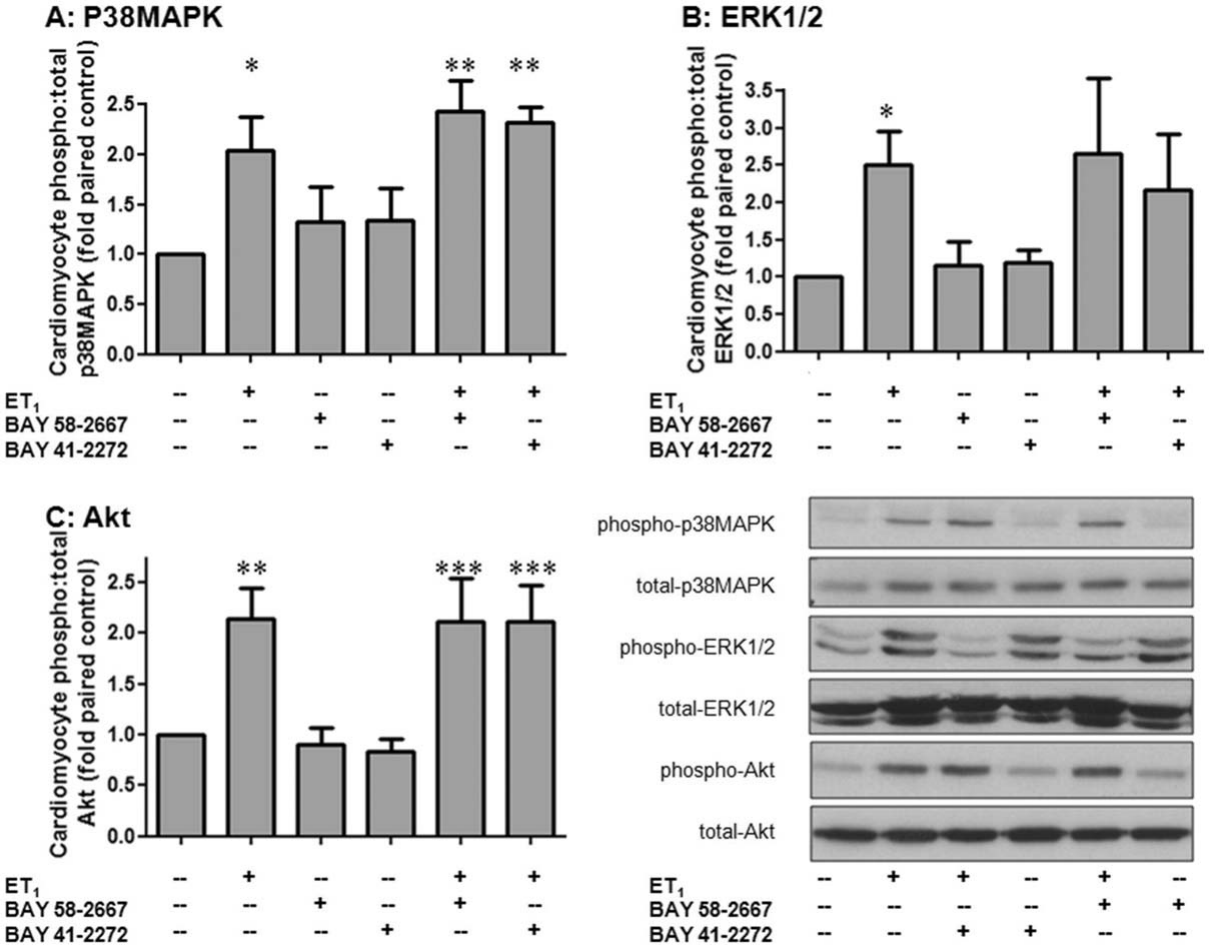
Impact of BAY 58-2667 on cardiomyocyte signal transduction. The effects of BAY 58-2667 on ET_1_-stimulated phosphorylation of the cardiomyocyte growth signals **A** p38MAPK, **B** ERK1/2 and **C** Akt. Results for BAY 41-2272 are shown for comparison. Both sGC ligands were present at 0.1 µmol/L for 48 h, with ET_1_-added for the final 10 mins (n = 8 cardiomyocyte preparations). Values are expressed as fold of paired control myocytes and given as mean±SEM, where n = number of myocyte preparations. *P<0.05, **P<0.01 and ***P<0.001vs control (one-way repeated measures ANOVA with Student-Newman-Keuls *post-hoc* analysis).

### Isolation of primary neonatal rat cardiomyocytes and fibroblasts

All materials used for cardiomyocyte isolation were of tissue culture grade. Cardiomyocytes were isolated from neonatal (1–2 day old) Sprague-Dawley rats using serial enzymatic digestion as previously described [14], [15], [27], [28]. Cardiomyocytes were suspended in sterile Dulbecco's Modified Eagle's Medium (DMEM), supplemented with penicillin 100 U/mL, streptomycin 100 μg/mL and 10% fetal calf serum (FCS). The cells were pre-plated twice (45 min at 37°C) to reduce fibroblast contamination, prior to plating at a low density of 2×10^4^cells/cm^2^ for measurement of cardiomyocyte size (13 mm round coverslips, to permit delineation of single, not overlapping, cells) and at high density of 1×10^5^cells/cm^2^ (∼90–95% confluence) for determination of all other responses. The cardiomyocyte culture medium was changed to serum-free DMEM after 48 h and cells were then incubated at 37°C for 48 h prior to treatment. The remaining attached cells following the two pre-plating steps (cardiac fibroblasts) were cultured in DMEM supplemented with 10% FCS and grown to confluence. Cardiac fibroblasts were then plated at 75,000 cells/ml, reaching 50% confluence after 24 h at 37°C. The fibroblast culture medium was then changed to serum-free DMEM for a further 24 h prior to treatment. Primary fibroblasts up to passage level 2 were used for experiments.

**Figure 5:**
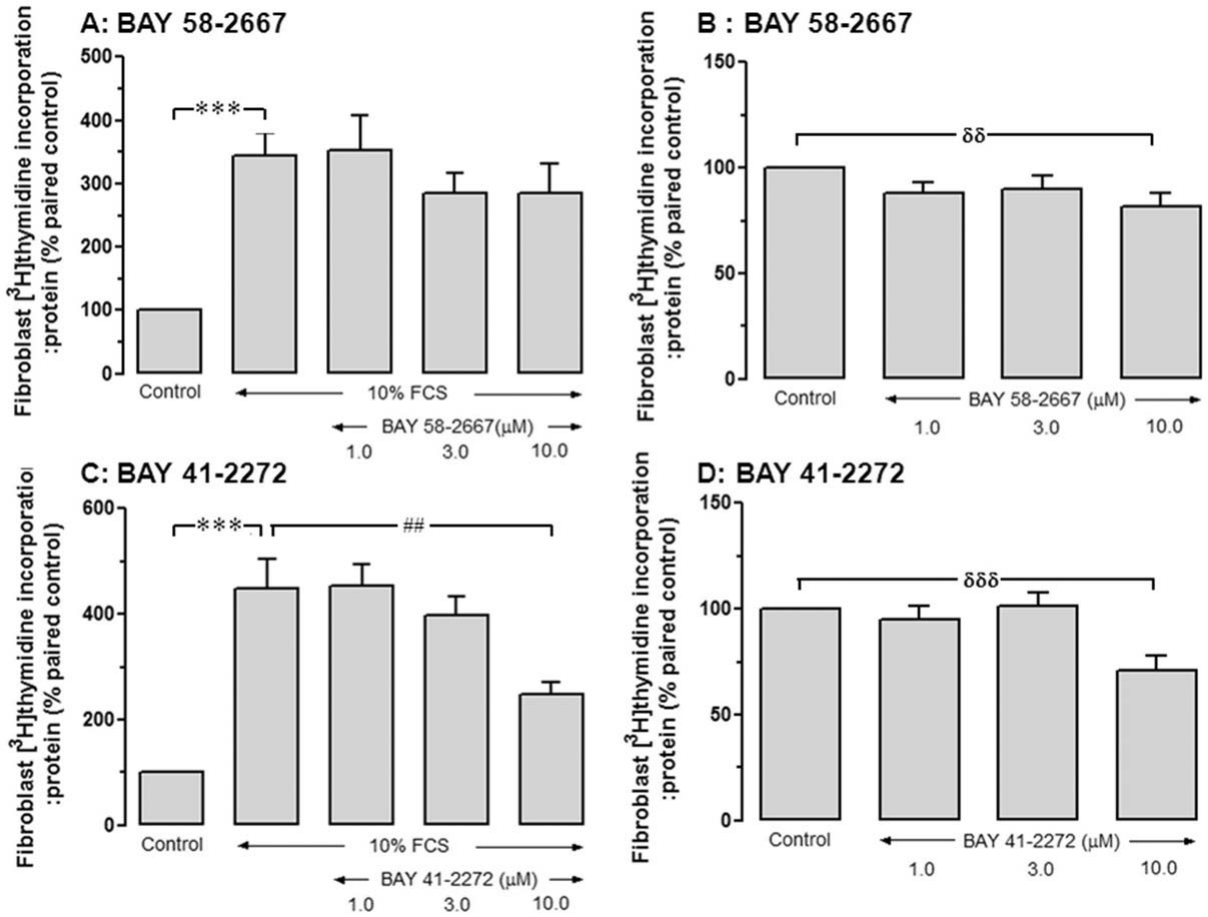
High concentrations of BAY 58-2667 are required for antiproliferative actions in cardiac fibroblasts. The effects of BAY 58-2667 on **A** FCS-stimulated and **B** basal DNA synthesis in cardiac fibroblasts (1–10 µmol/L, n = 6–9 fibroblast preparations). The effects of BAY 41-2272 on **C** FCS-stimulated and **D** basal DNA synthesis in cardiac fibroblasts (1–10 µmol/L, n = 5–8 fibroblast preparations). ***P<0.001, ^δδδ^P<0.001, ^δδ^P<0.01 vs control, ^##^P<0.01 vs 10% FCS alone (one-way ANOVA with Bonferroni *post-hoc* analysis).

### Cardiomyocyte hypertrophic responses

Cardiomyocytes were incubated in the presence and absence of the hypertrophic stimulus endothelin-1 (ET_1_, 60 nmol/L) and BAY 58-2667 or BAY 41-2272 (both 0.01–0.3 µmol/L). BAY 58-2667 and BAY 41-2272 were provided by Bayer Schering Pharma AG (Germany). Vehicle controls were also performed (0.0165% DMSO). Cardiomyocyte hypertrophy was measured as two-dimensional (2D) area of individual cells, or *de novo* protein synthesis, as described previously [15], [29]. Briefly, following 48 h incubation with study drugs (ET_1_ ± BAY 58-2667 or BAY 41-2272), coverslips plated with cardiomyocytes at low density were inverted onto microscope slides. Using a phase contrast microscope at 1034×1300 resolution, 6–8 fields of live myocytes were randomly chosen and photographed at 10× magnification. Images were analysed using Optimas software (Media Cybernetics, Silver Springs, USA), with a photographed scale of the 10× objective lens for calibration. The 2D area (μm 2) of cardiomyocytes was calculated by tracing edges of live cells. For each treatment, 30–40 individual myocytes were measured [15], [29]. Cardiomyocyte *de novo* protein synthesis was determined via [^3^H]phenylalanine incorporation and normalised to DNA content, with 3 replicates per treatment) at completion of 48 h drug treatment, as described previously [15], [28], [15,29]. Myocytes plated at high density were incubated for 48 h with [^3^H]phenylalanine (2 µCi/mL; Amersham Biosciences, Castle Hill, Australia) with or without study drugs (ET_1_ ± BAY 58-2667 or BAY 41-2272). Protein and DNA were then precipitated with 10% trichloroacetic acid. The precipitate was washed with 95% ethanol and the resulting pellet resuspended in 0.15 mol/L sodium hydroxide. [^3^H]Phenylalanine incorporation was determined by scintillation counting an aliquot of each sample. Results were normalized to nanograms of DNA/well to correct for cell number [15], [29].

**Table 2:** The effect of lower concentrations of BAY 58-2667 (0.1–0.3 µmol/L) on basal and FCS-stimulated cardiac fibroblast DNA synthesis.

	Control	10% FCS	BAY 58-2667 (0.1µmol/L) +10% FCS	BAY 58-2667 (0.3µmol/L) +10% FCS
FCS-stimulated [^3^H]thymi-dine incorporation (%)	100±0%	367±49%*	352±44%	369±44%

	**Control**		**BAY 58-2667 (0.1µmol/L)**	**BAY 58-2667 (0.3µmol/L)**
Basal [^3^H]thymidine incorporation (%)	100±0%		95±5%	91±4%

Values are expressed as percentage of paired control fibroblasts and given as mean±SEM, n = 5–10 per group. *P<0.05 vs control (one-way ANOVA with Bonferroni *post-hoc* test).

**Table 3:** The effect of lower concentrations of BAY 41-2272 (0.1–0.3 µmol/L) on basal and FCS-stimulated cardiac fibroblast DNA synthesis.

	Control	10% FCS	BAY 41-2272 (0.1µmol/L) +10% FCS	BAY 41-2272 (0.3µmol/L) +10% FCS
FCS-stimulated [^3^H]thymi-dine incorporation (%)	100±0%	432±63%***	382±75%	380±49%

	**Control**		**BAY 41-2272 (0.1µ mol/L)**	**BAY 41-2272 (0.3µ mol/L)**
Basal [^3^H]thymidine incorporation (%)	100±0%		101±5%	92±4%

Values are expressed as percentage of paired control fibroblasts and given as mean±SEM, n = 5-10 per group. ***P<0.001 vs control (one-way ANOVA with Bonferroni *post-hoc* test).

### Cardiomyocyte superoxide generation

Superoxide generation is a key trigger of cardiomyocyte hypertrophic responses [15], [30]; this was determined using NADPH-driven lucigenin-enhanced chemiluminescence, as previously described [15], [30]. Cells were incubated for 24 h with ET_1_, in the presence and absence of BAY 41-2272, BAY 58-2667 (both 0.1 µmol/L) or DMSO vehicle. sGC ligands were present for 24 h prior to, and for the duration of ET_1_ incubation. Media was replaced with a Krebs-HEPES buffer containing NADPH (100 μmol/L) and lucigenin (5 μmol/L) for chemiluminescence measurements, determined as relative light units per second (RLU/sec). Background luminescence (in the absence of cells) was subtracted from the average of 8 readings. Each experiment was studied with at least 6 replicates and the average result was taken.

**Figure 6:**
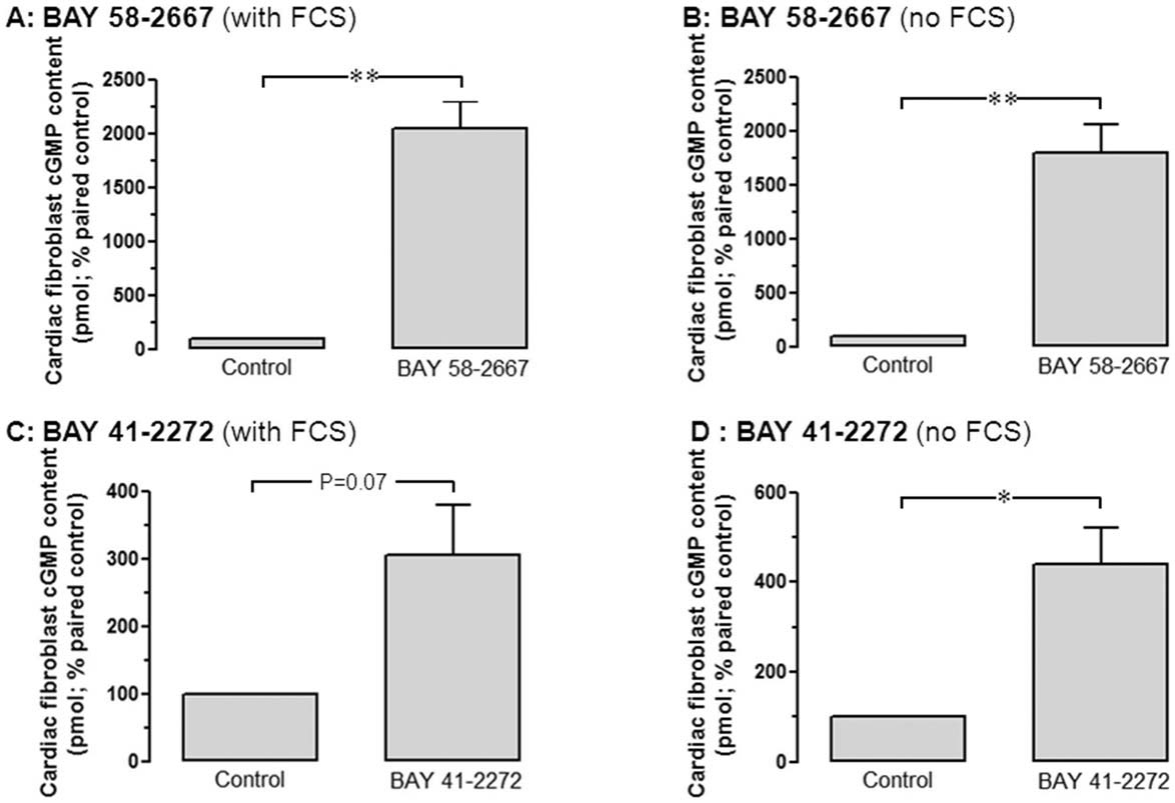
BAY 58-2667 elevates cardiac fibroblast cGMP content. BAY 58-2667 significantly increases cardiac fibroblast cGMP accumulation, both **A** in the presence of 10% FCS, and **B** in the absence of FCS (both 3.0 µmol/L, n = 4 fibroblast preparations). Similar results were obtained for BAY 41-2272, both **C** in the presence of 10% FCS, and **D** in the absence of FCS (both 3.0 µmol/L, n = 4 fibroblast preparations). *P<0.05, **P<0.01 vs control (Student's paired *t-*test).

### Cardiomyocyte signal transduction

In a separate series of experiments, the impact of sGC activation on intracellular signal transduction including phosphorylation of the mitogen-activated protein kinases p38MAPK (implicated in pathological hypertrophy) and ERK1/2, as well as Akt (a cell survival kinase Akt) was determined. Cardiomyocytes were incubated for 48 h in the presence and absence of sGC ligands (both 0.1 µmol/L) or DMSO vehicle. ET_1_ was added only for the final 10 min. Western analyses used phospho-specific antibodies (Cell Signaling Technology, Danvers, MA), as previously described [14], [31], [32].

**Figure 7:**
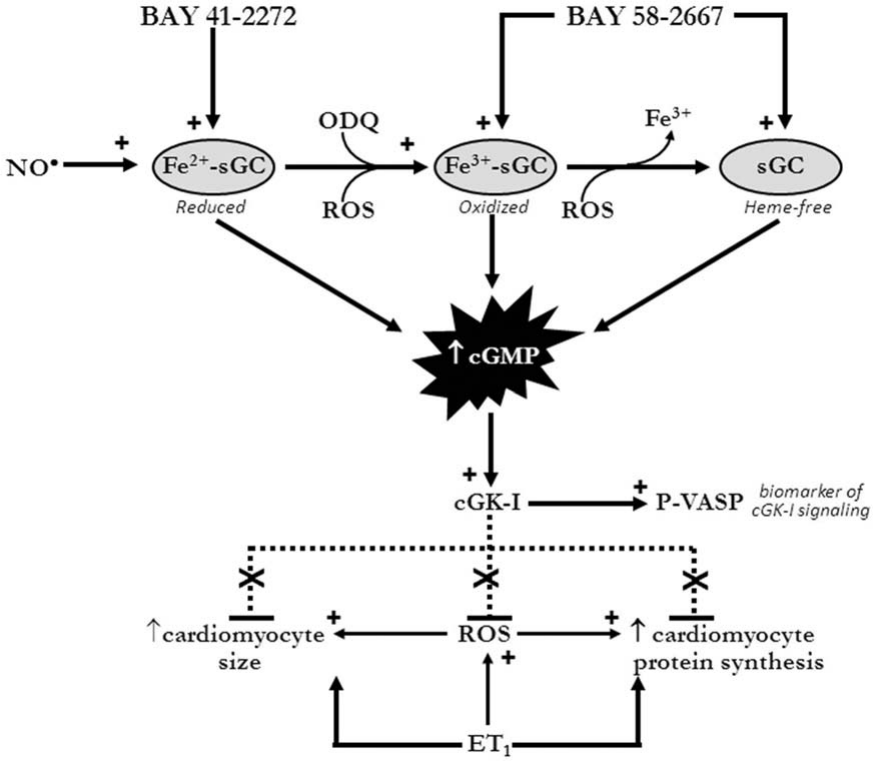
Mechanism of antihypertrophic actions of sGC ligands in cardiomyocytes. Our findings indicate that the cardiomyocyte actions of the sGC activator BAY 58-2667 are mediated via sGC/cGMP/cGK-I signaling, and are potentiated by the sGC oxidizing agent ODQ, implicating a role for oxidized sGC in these actions. This is distinct to both NO·, and the NO·–independent sGC stimulator BAY 41-2272, which utilize reduced sGC/cGMP/cGK-I signaling to suppress the hypertrophic response (including cell size and *de novo* protein synthesis) and its key trigger, cardiomyocyte superoxide. No role for cAMP-mediated actions of either sGC ligand was observed. See text for references.

### Cardiac fibroblast proliferation

DNA synthesis, an *in vitro* marker of cardiac fibrosis, was measured via [^3^H]thymidine incorporation (3 replicates per treatment) as previously described [33], with some modification. Cardiac fibroblasts were treated for 24 h with BAY 41-2272 (0.1–10 µmol/L) or BAY 58-2667 (0.1–10 µmol/L), in the absence or presence of 10% FCS, for determination of basal and stimulated cell proliferation, respectively. DMSO vehicle controls were also included. Cells were then incubated for a further 2 h at 37°C in serum-free DMEM containing [^3^H]thymidine (2 µCi/mL, MP Biochemical, Solon OH USA). Cardiac fibroblast protein and DNA were trichloroacetic acid–precipitated before resuspension in 1.0 mol/L NaOH. [^3^H]Thymidine incorporation was determined by liquid scintillation, and normalized to protein content.

### cGMP/cAMP signaling

To measure cardiomyocyte cGMP and cAMP accumulation, cardiomyocytes were incubated with BAY 41-2272, BAY 58-2667 (both 0.1 µmol/L) or DMSO vehicle for 15 minutes in DMEM containing 1 mmol/L 3-isobutyl-1-methylxanthine (IBMX). In separate experiments, the impact of the sGC oxidizing agent, 1H-[1], [2], [4]oxadiazolo[4,3-a]quinoxalin-1-one (ODQ, 10 μmol/L) on sGC ligand cardiomycoyte cGMP accumulation was also determined. To measure cardiac fibroblast cGMP accumulation, fibroblasts were incubated with BAY 41-2272, BAY 58-2667 (both 3 µmol/L) or DMSO vehicle for 15 minutes in DMEM containing 1 mmol/L IBMX, in the absence or presence of 10% FCS. According to manufacturer's instructions, cell-lysates were then snap frozen in liquid nitrogen and stored at −80°C until enzyme-immunoassay (Cayman Chemical Company, Ann Arbor MI, USA). In separate experiments, cardiomyocytes were incubated with BAY 41-2272, BAY 58-2667 (both 0.1 µmol/L) or DMSO vehicle for 10 minutes for Western analysis of vasodilator-stimulated phosphoprotein (VASP)-phosphorylation at serine 239 (VASP-P^ser239^) using phospho-specific antibodies (Cell Signaling Technology, Danvers, MA), as previously described [31], [32].

### Statistical analysis

All results were expressed as mean ± standard error for each treatment group, with *n* denoting the number of cell preparations. All measures were expressed as a percentage of paired control cardiomyocytes or cardiac fibroblasts. Statistical differences were analyzed using one way repeated measures analysis of variance (ANOVA, with Bonferroni or Student Newman-Keuls *post-hoc* analysis for multiple comparison of normally- or non-normally-distributed data, respectively), or Student's paired *t-*test, as appropriate (using Graphpad Prism 5.0). P*<*0.05 was accepted as significant.

## Results

### BAY 58-2667 elicits potent antihypertrophic actions in neonatal cardiomyocytes

ET_1_ (60 nmol/L) induced a hypertrophic response in neonatal rat cardiomyocytes, on both computer-aided analysis of 2D cardiomyocyte area (by 39±4%, from 1280±50 to 1780±81 µm^2^, n = 9 cardiomyocyte preparations, P<0.0001) and *de novo* protein synthesis (by 31±6%, from 730±183 to 911±202 counts/ng DNA, n = 10 P<0.0005). BAY 58-2667 elicited concentration-dependent inhibition of the ET_1_-induced increase in cell-size, such that after 48 h, 0.3 µmol/L BAY 58-2667 decreased 2D area from 137±3% to 108±7% of paired control (P<0.001, Figure 1A). BAY 41-2272 exhibited a similar concentration-dependent inhibition of ET_1_-induced increase in cell-size, with 0.3 µmol/L BAY 41-2272 reducing 2D area from 146±9% to 108±6% of paired control (P<0.001, Figure 1B). Representative results in individual cardiomyocytes are shown in Figure 1C. BAY 58-2667 also significantly attenuated ET_1_-stimulated protein synthesis over the same concentration range (although these were not concentration-dependent). For example, after 48 h, 0.3 µmol/L BAY 58-2667 decreased cardiomyocyte protein synthesis from 141±11% to 106±16% of paired control (P<0.05, Figure 1D). In comparison, BAY 41-2272 (0.1 µmol/L) similarly attenuated ET_1_-stimulated protein synthesis from 122±3% to 102±7% of paired control (P<0.05, Figure 1E), although the effect of BAY 41-2272 may have been lost at 0.3 µmol/L. Importantly, DMSO (0.0165%) vehicle alone had no significant effect on either cardiomyocyte size or protein synthesis (95±6% and 105±14% of paired control cardiomyocytes, respectively, both n = 5).

### BAY 58-2667 potently activates cardiomyocyte cGMP/P-VASP signaling

As expected for a selective sGC activator, BAY 58-2667 (0.1 µmol/L) markedly potentiated cGMP/P-VASP signaling, elevating cardiomyocyte cGMP to 308±50% of paired control (P<0.01; Figure 2A) and its downstream signal, phosphorylation of VASP, a marker for cGMP-dependent protein kinase cGK-1 activity to 236±38% that of paired control (P<0.05; Figure 2B). At the same concentration, BAY 41-2272 also stimulated cardiomyocyte cGMP accumulation (to 173±12% paired control, P<0.01; Figure 2C) and VASP-phosphorylation (to 153±21% paired control, P* = *0.05; Figure 2D), but was less potent than BAY 58-2667. In contrast, neither BAY 58-2667, BAY 41-2272 nor DMSO vehicle significantly affected cardiomyocyte cAMP accumulation from paired control, although BAY 41-2272 exhibited a non-significant trend for modestly reduced cAMP accumulation (Table 1).

### BAY 58-2667 suppresses cardiomyocyte ROS

ROS such as superoxide act as a key trigger of the hypertrophic response in cardiomyocytes [15], [30], yet these species may oxidize sGC rendering it insensitive to NO^•^ [34], [35] and possibly to BAY 41-2272 [23]. We thus sought to determine whether BAY 58-2667 suppressed superoxide generation, whether its ability to increase sGC activity is affected by the sGC oxidizing agent ODQ. ET_1_ (60 nmol/L) significantly increased NADPH-derived cardiomyocyte superoxide generation, to almost 2-fold paired control (P<0.001), which was sensitive to BAY 58-2667 but not to BAY 41-2272 (both 0.1 µmol/L, Figure 3A and 3B). Furthermore, pre-treatment of cardiomyocytes with ODQ (10 µmol/L) increased cGMP accumulation in response to BAY 58-2667 9-fold (P<0.001; Figure 3C). Conversely, ODQ did not affect BAY 41-2272-stimulated cGMP generation (Figure 3D, P = NS), DMSO alone had no effect on cardiomyocyte superoxide generation or cGMP generation (results not shown).

### Impact of BAY 58-2667 on cardiomyocyte signal transduction

ET_1_ acutely activated a spectrum of cardiomyocyte pro-growth signals in cardiomyocytes, including p38MAPK (by 2.0±0.3-fold, n = 4 cardiomyocyte preparations, P<0.05, Figure 4A), ERK1/2 (by 2.5±0.4-fold, n = 6 cardiomyocyte preparations, P<0.05, Figure 4B) and Akt (by 2.1±0.3-fold, n = 5 cardiomyocyte preparations, P<0.01, Figure 4C). Interestingly, pretreatment with BAY 58-2667 (0.1 µmol/L) for 48 h did not impact on the effect on either basal or ET_1_-stimulated kinase phosphorylation; similar evidence was observed with the same concentration of BAY 41-2272 (Figure 4). DMSO alone had no effect on basal or ET_1_-stimulated kinase phosphorylation (results not shown).

### Supra-pharmacological BAY 58-2667 concentrations are required for antifibrotic actions *in vitro*

The proliferative response to 10% FCS in neonatal cardiac fibroblasts was evident on elevated fibroblast DNA synthesis to 4-fold paired control (P*<*0.001; Table 2). Yet at concentrations efficacious in cardiomyocytes (0.1–0.3 µmol/L), BAY 58-2667 failed to inhibit either FCS-stimulated or basal DNA synthesis (Table 2). Higher concentrations of BAY 58-2667 (10 µmol/L) only modestly tended to reduce FCS-stimulated cardiac fibroblast DNA synthesis, from 3.4±0.4-fold to 2.9±0.5-fold control (n = 7, P = NS, Figure 5A), although basal DNA synthesis was significantly reduced (to 82±6% paired control, P*<*0.01; Figure 5B). Similarly, BAY 41-2272 affected neither FCS-stimulated or basal DNA synthesis synthesis in cardiac fibroblasts over 0.1–0.3 µmol/L (Table 3). The addition of 10 µmol/L BAY 41-2272 however significantly attenuated both stimulated and basal levels of fibroblast proliferation (P*<*0.01, Figure 5C and P *<*0.001; Figure 5D, respectively). Of note, DMSO vehicle for these sGC ligands did not significantly affect FCS-stimulated or basal DNA synthesis (3.4±0.7-fold and 1.1±0.4-fold paired control, both P = NS, respectively).

### BAY 58-2667 elevates fibroblast cGMP

As was observed in cardiomyocytes, BAY 58-2667 (3 µmol/L) was a particularly potent generator of cardiac fibroblast cGMP, elevating it to 20-fold paired control in the presence of 10% FCS (P<0.01, Figure 6A), and 18-fold paired control in the absence of FCS (P<0.01, Figure 6B). In the presence of FCS, BAY 41-2272 (3 µmol/L) tended to elevate fibroblast cGMP accumulation (to 3.1±0.7-fold paired control, P = 0.07), which became significant in the absence of FCS (where fibroblast cGMP levels were increased to 4.4±0.8fold paired control, P<0.05 Figure 6C and 6D).

## Discussion

This study demonstrated that the sGC activator BAY 58-2667 elicits cardioprotective effects *in vitro*, limiting cardiomyocyte hypertrophy. Our findings represent the first scientific evidence that these antihypertrophic actions are evident in the absence of confounding hemodynamic factors, are manifest at low (submicromolar) concentrations and are associated with cGMP signaling. In addition, we show that BAY 58-2667 only modestly inhibits cardiac fibroblast proliferation *in vitro* and elevates fibroblast cGMP, but only at suprapharmacological concentrations. The actions of BAY 58-2667 were mimicked by the sGC stimulator, BAY 41-2272. Our data suggests the possibility of cardiomyocyte-selective activity of these sGC ligands.

In the current study, ET_1_ increased both markers of cardiomyocyte hypertrophy, 2D area and *de novo* protein synthesis, in accordance with previous findings [15], [36]. BAY 58-2667 potently abrogated these effects. Chronic *in vivo* treatment with either BAY 58-2667 or BAY 41-2272, using relatively high doses of sGC ligand, has been shown previously to limit left ventricular hypertrophy in a range of hypertension-driven experimental models [16]–[20]. In each of these settings however, the antihypertrophic effect appeared secondary to the attenuation of hypertension via vasodilatation. Thus our findings are the first to demonstrate a direct cellular antihypertrophic effect of these ligands, independent of confounding hemodynamic factors. The proposed mechanism of these effects is illustrated in Figure 7.

We have shown previously that both NO^•^/cGMP signaling via the activation of sGC, and natriuretic peptide/pGC/cGMP signaling, are powerful antihypertrophic mechanisms in both adult and neonatal rat cardiomyocytes, as well as the intact heart [8], [10], [12]–[15], [29]. The majority of *in vitro* studies addressing cardiomyocyte hypertrophy have chosen the neonatal phenotype we have used here. Our own previous studies have confirmed cGMP-dependent antihypertrophic properties of both NO donors and natriuretic peptides are also observed in the adult context [10], [12], [28], [29], where hypertrophic responses can only be studied over a much shorter time-frame, 2 h (in contrast to the longer-term 48 h time-frame possible with the neonatal phenotype. This shorter time-frame would preclude assessment of changes in cell size. From the current study, it is readily apparent that the antihypertrophic effects of BAY 58-2667 are mediated via similar mechanisms to other cGMP-elevating agents, but at much greater potency, given its ability at 0.1 µmol/L to directly elevate both cardiomyocyte cGMP content and VASP phosphorylation, downstream of cGK-1 [37]. Of note, although BAY 58-2667 and BAY 41-2272 demonstrated near equal potency in their antihypertrophic activities, BAY 58-2667 was more effective at increasing cardiomyocyte cGMP content. We have observed previously similar discrepancies between the degree of elevation of cGMP content and the subsequent extent of functional effects in response to cGMP-elevating agents (particularly NO^•^ donors), in both rat aortae [38] and neonatal rat cardiomyocytes (Irvine *et al*, unpublished observations), suggesting that very low amounts of cGMP are sufficient for full biological response to sGC agonists [39].

As illustrated in Figure 7, our findings also support the potential presence of pools of endogenously-oxidized/heme-free sGC in untreated cardiomyocytes, which are sensitive to BAY 58-2667 yet insensitive to BAY 41-2272 [24], [26]. Heme oxidation and heme release in only a small proportion of sGC would likely result in a significant elevation of BAY 58-2667-induced sGC activity [40]. In support of this, our demonstration that treatment with the sGC-oxidizing agent ODQ had a marked impact on BAY 58-2667 in cardiomyocytes, such that cGMP levels were increased 9-fold versus the sGC ligand alone, yet had no impact on the response to BAY 41-2272. Such potentiation of the response to BAY 58-2667 following ODQ is in agreement with previous observations in cultured pulmonary artery smooth muscle [41] and endothelial cells [42], and demonstrate that the potency of BAY 58-2667 is dramatically increased when sGC has been oxidized. In contrast, ODQ surprisingly failed to suppress BAY 41-2272-stimulated cardiomyocyte cGMP levels. ODQ potently blunts sGC activity in immortalized cell lines [42], [43] and platelets [44], in addition to impairing BAY 41-2272-stimulated vaso- and broncho-relaxation *in vitro* [45], [46]. The stimulatory effect of 0.1 µmol/L BAY 41-2272 on cardiomyocyte cGMP levels was relatively modest, likely a result of the low concentration used. Although our findings of minimal impact of ODQ on BAY 41-2272-stimulated cGMP levels in cardiomyocytes contrast to the impact of ODQ on vascular cGMP content stimulated by higher concentrations of BAY 41-2272 [45], they are however consistent with previous observations in primary endothelial cells [42], where the availability of a reserve of intracellular sGC was postulated. As such, the mechanism for the lack of inhibition of ODQ on BAY 41-2272-stimulated cardiomyocyte cGMP content warrants further investigation.

ROS generation plays a major causative role in the cardiac hypertrophic response both *in vivo* and *in vitro* [8], [10], [12]–[15], [47]. Indeed previous studies from our laboratory have demonstrated that the ROS-suppressing effects of the cGMP-elevating natriuretic peptides, or cGMP itself contribute, at least in part, to their antihypertrophic effects in cardiomyocytes [14], [15]. We now demonstrate that the sGC ligand BAY 58-2667 (but not BAY 41-2272) suppresses ET_1_-stimuluated cardiomyocyte superoxide content. This selective efficacy of BAY 58-2667 may reflect a potentially greater pool of endogenously-oxidized sGC in our cardiomyocyte preparation, and highlights a further advantage of BAY 58-2667's potential for cardioprotective effects. Whether BAY 58-2667 also activates other, cGMP-independent signaling remains a topic of debate. We also observed that BAY 58-2667 did not alter cardiomyocyte cAMP. The anti-aggregatory activity of BAY 58-2667 in platelets has been attributed to cGMP generation, with a secondary increase in cAMP [48]. Yet in contrast in the heart, BAY 58-2667 only increased cGMP (and not cAMP), in agreement with our observations [49]. Given that p38MAPK is a ROS-sensitive kinase that contributes to pathological hypertrophy [50], it was thus unexpected that BAY 58-2667 did not alter their cardiomyocyte activity in the current study. Given the cardioprotective properties of Akt (and possibly also ERK1/2) as cell survival kinases [8], [51], it is highly favourable that BAY 58-2667 so potently inhibits cardiomyocyte hypertrophy and its key trigger superoxide, yet preserves protective cardiomyocyte signaling. Our finding that BAY 58-2667 blunts one mediator of pathological hypertrophy (superoxide) yet leaves two mediators of cell survival intact (Akt, ERK1/2) is an attractive trait. It is possible that by assessing pathological p38MAPK activation at a single timepoint (10 mins after ET_1_), we missed potential BAY 58-2667 actions on this kinase. BAY 58-2667 convincingly activated sGC and cGK-1 in our cardiomyocytes (on cGMP content and VASP phosphorylation); perhaps insufficient time was provided for cGK-I to upregulate MAPK phosphatase-1 (MKP-1) and hence to dephosphorylate p38MAPK. Thus the mechanisms by which direct sGC activation inhibits cardiomyocyte hypertrophy distal to suppressed ROS generation warrant further investigation.

Cardiac remodeling in the intact heart comprises contribution from both pathological hypertrophy and the development of cardiac fibrosis. Increased fibroblast proliferation and extracellular matrix production increases myocardial stiffness, further impairing systolic and diastolic function [52], and subsequent progression to heart failure. To our knowledge, our study is the first to investigate the potential for an sGC activator to affect cardiac fibrosis. Previous studies have shown that chronic treatment with sGC stimulators such as BAY 41-2272 or BAY 63-2521) at doses of 10 mg/kg/day inhibited a range of pro-fibrotic markers in renal fibrosis [53], [54], as well as hypertension-induced cardiac fibrosis [20], [55]; these antifibrotic effects were all secondary to reductions in blood pressure. Lower, subpressor doses of BAY 41-2272 (2 mg/kg/day) however have been shown to also elicit modest antifibrotic effects in settings of pressure-overload *in vivo* [20], [56]. Under our experimental conditions however, we were unable to demonstrate a robust effect of either sGC ligand against fibroblast proliferation. Only suprapharmacological concentrations of BAY 41-2272 (10 µmol/L) significantly inhibited FCS-stimulated thymidine incorporation, with BAY 58-2667 virtually lacking any antiproliferative effects under these conditions. Further, both sGC ligands only reduced basal fibroblast proliferation by ∼20–30% even at the highest concentration studied (10 µmol/L) of BAY 41-2272 or BAY 58-2667, which is in contrast to the more marked effects previously reported [20]. Whether reduced (rather than oxidized) sGC predominated in our cultured cardiac fibroblasts was not specifically investigated in the present study; interestingly, the less potent antiproliferative effects of both sGC ligands in our hands were however not a result of a reduced ability to stimulate fibroblast cGMP.

### Perspectives and conclusions

In summary, these data provide evidence that BAY 58-2667 elicits cardioprotective effects *in vitro*, limiting cardiomyocyte hypertrophy. These apparent cardiomyocyte-selective actions are associated with sGC activation and are evident in the absence of confounding hemodynamic factors, at low (submicromolar) concentrations. Whether the apparent selectivity for antihypertrophic versus antifibrotic effects of BAY 58-2667 is a feature unique to healthy myocardium (whereas the antifibrotic effects also become important in chronic disease scenarios, where sGC may be oxidized and/or heme-free), remains to be determined. Thus, this distinctive NO^•^-independent sGC activator, which exploits sGC/cGMP-dependent signaling, may represent innovative pharmacotherapy for limiting myocardial hypertrophy in clinical settings.
